# Molecular Dynamic Simulations of the Physical Properties of Four Ionic Liquids

**DOI:** 10.3390/ijms252011217

**Published:** 2024-10-18

**Authors:** Jing Fan, Yuting Pan, Zhiqiang Gao, Hongwei Qu

**Affiliations:** School of Energy and Power Engineering, Northeast Electric Power University, Jilin 132012, China; crystalfan@neepu.edu.cn (J.F.); 2202300434@neepu.edu.cn (Y.P.);

**Keywords:** novel solvents, ionic liquids, physical properties, density, viscosity, thermal conductivity, RNEMD, NEMD

## Abstract

In this study, the molecular structure models of four ionic liquids were created, the reverse nonequilibrium molecular dynamics simulation (RNEMD) approach was used to predict their densities and viscosities, and their thermal conductivity was simulated using the non-equilibrium molecular dynamic simulation method (NEMD). The calculated results of ionic liquid densities were compared with the data in the literature; most of the variances are around 2.5%, and the maximum relative deviation was less than 6.27%; viscosity values were compared with the experimental data, with a maximum relative deviation of −8.96% and a minimum relative deviation of −2.72%. The simulated thermal conductivity has a good linear relationship with respect to temperature and pressure, which is in good agreement. This study provides a reference for molecular dynamics simulation to measure the physical properties of ionic liquids, which is important for the development of ionic liquids.

## 1. Introduction

In recent years, the global energy situation has become more and more critical as humankind has become overly dependent on fossil fuels. Additionally, the excessive emissions of combustion byproducts, such as greenhouse gasses and inhalable particulate matter, during the process of using fossil fuels have seriously harmed the environment and contributed to global warming. The creation of new alternative working mediums has become a hot topic in light of the escalating energy crisis and global climatic issues. Inorganic or organic cations and inorganic or organic anions are combined to form ionic liquids, which are liquid salts at or close to room temperature. The advantages of ionic liquid over typical heat transfer fluids including water, ethylene glycol, molten salt, silicone oil, synthetic oil, and mineral oil include a wide liquid range, low steam pressure, good chemical and thermal stability, and their recyclability. Additionally, the use of ionic liquids as new heat transfer fluids has the potential to not only considerably improve heat transfer efficiency but also to significantly reduce the size of the individual parts of heat transfer equipment [1,2].

The physical properties of ionic liquids are important for scaling their application. However, the abundance of ionic liquids has created the issue of suiting their properties, which are determined through experimental measurement, to their requirements. The shortcomings of a lengthy experimental period and expensive experimental equipment can be overcome by the molecular dynamics simulation method. Different forms of novel ionic liquids can be quickly generated by altering the type of anion and cation or altering the associated groups, significantly reducing the development time of ionic liquids. In order to model the thermophysical characteristics of ionic liquids, such as density and viscosity, for the purpose of altering their structure and improving their properties, a huge number of researchers have turned to molecular simulation techniques in recent years. Małgorzata et al. [3] established a non-polarized molecular force field model of [Emim][B (CN)_4_] through the optimization of experimental data. Liu et al. [4] explored the specific heat capacity, density, self-diffusion coefficient, viscosity coefficient and thermal conductivity of six ionic liquids including [Bmim][TF_2_N], and [Bmim][PF_2_N] by experimental measurement and molecular dynamics simulation, respectively. Goloviznina et al. [5] improved the CL&Pol polarization model for ionic liquids, improving on the previous generation of fixed-charge models, which has contributed to the development of the field and the design of better ionic liquids. Dmitry et al. [6] reviewed the advantages and disadvantages of applying different polarization treatments for ionic liquids, use the mean-field method and explicit polarization models commonly used in molecular dynamics simulation of ion materials to study ionic liquid properties, discuss the advantages and disadvantages of these two methods, and propose strategies for developing polarization models. Wang et al. [7] constructed an all-atom structure model of ionic liquid 1-n-butyl-3-methyl imidazole double (trifluoromethylsulfonyl) imide ([Bmim][Tf_2_N]), and conducted molecular dynamics simulation of its density and system interaction energy at five temperatures and five pressures.

The equilibrium molecular dynamics (EMD) technique and non-equilibrium molecular dynamics (NEMD) method are two general categories into which the simulation system may be categorized when calculating thermal characteristics using molecular simulation [8,9]. The NEMD method has better convergence, and is widely used in property simulation [10,11,12]. RNEMD is an isotropic NEMD method developed based on the Müller-Plathe [13] theory. This method involves creating a shear force field in a simulated system that interacts non-physically. The flow layer is artificially divided into various regions in the simulation system, and at each particular time step, the momentum of the particles in the region is exchanged, realizing the creation of a velocity gradient and the subsequent formation of a shear force field. This approach can be quickly calculated and is more precise in simulating actual fluid flow conditions. Therefore, in this paper, the RNEMD method is used to calculate the density and viscosity, and the NEMD method is used to calculate the thermal conductivity of the four ionic liquids. The relationships of density, viscosity, and thermal conductivity with pressure and temperature are analyzed. And the simulation results are compared with the experimental or literature values.

## 2. Results and Discussion

### 2.1. Density

**Figure 1:**
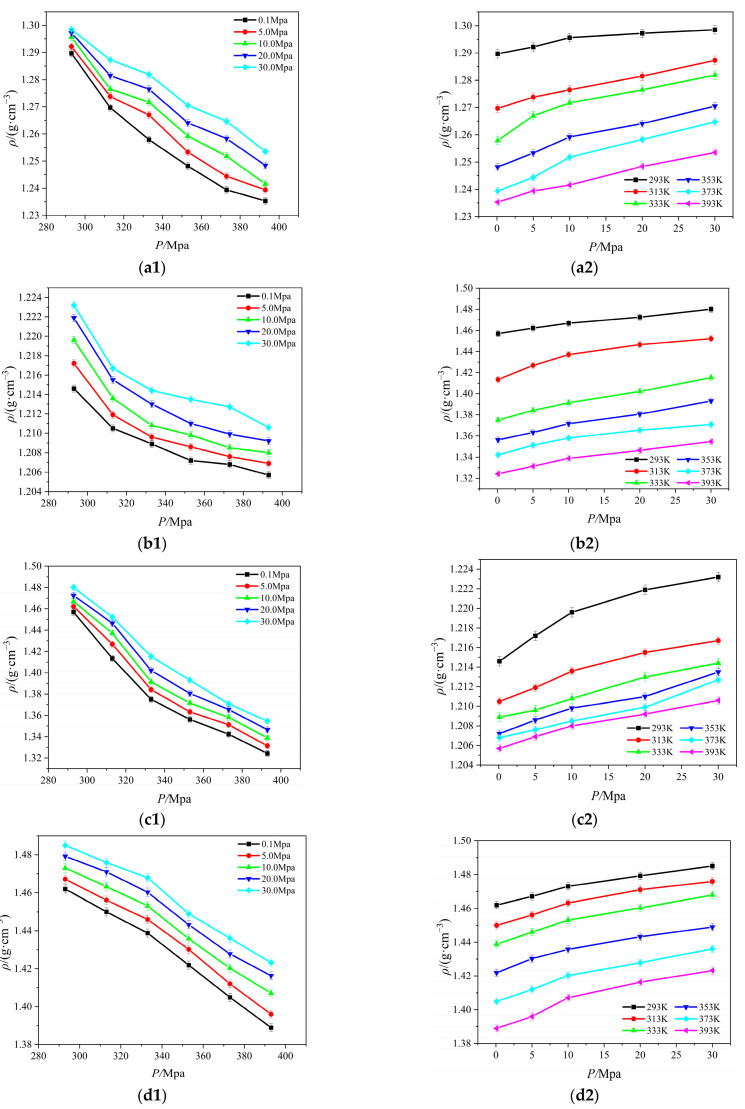
Relationship between four ionic liquid densities for temperature and pressure: (**a**) [Emim][BF_4_]; (**b**) [Bmim][BF_4_]; (**c**) [Bmim][PF_6_]; (**d**) [Bmim][Tf_2_N].

The density of ionic liquids serves as the foundation for their industrial applications and is a key reference for the computation and measurement of thermodynamic parameters like the viscosity and surface tension of ionic liquids, and it is a physical parameter that is easily influenced by external variables. For this work, the molecular models for [Emim][BF_4_], [Bmim][BF_4_], [Bmim][PF_6_], and [Bmim][Tf_2_N] were constructed, and the density of these four ionic liquids was calculated at a temperature range of 293 K to 393 K and a pressure range of 0.1 MPa to 30.0 MPa. The relationship between the density with temperature and pressure is shown in Figure 1. All images in this section were drawn using original software [14].

Clearly, the density of [Emim][BF_4_], [Bmim][BF_4_], [Bmim][PF_6_], and [Bmim][Tf_2_N] increases with increasing pressure and decreases with increasing temperature. In general, the relationship between density and temperature and pressure is linear. The density changes more slowly in response to changes in pressure than it does to changes in temperature. As temperature rises, ionic liquid particle kinetic energy rises, the thermal motion of molecules speeds up, attraction between particles weakens, the number of particles per unit volume falls, and density follows suit.

From the a1 and b1 graph in Figure 1, the density of [Bmim][BF_4_] is lower than [Emim][BF_4_] at the same temperature and pressure, and the former is relatively more sensitive to temperature, and the density gradually stabilizes after 333 K. The density of [Bmim][BF_4_] is lower than [Emim][BF_4_] at the same temperature and pressure, and the [Emim][BF_4_] is substantially more sensitive to temperature, according to the the a1 and b1 graph in Figure 1. This is because when the anions in the ionic liquid are the same and the cations are different, with the length of the cationic alkyl side chain, the single ionic liquid molecule occupies more space, the number of particles per unit volume decreases, and the density decreases when the temperature increases. A similar phenomenon is displayed in the (b), (c), and (d) graphs in Figure 1. When the cations are the same and the anions are different, the more atoms that are on the anions, the more atoms interact with one another, making the molecules of the ionic liquid more compact. As the pressure rises, the density also rises.

**Figure 2:**
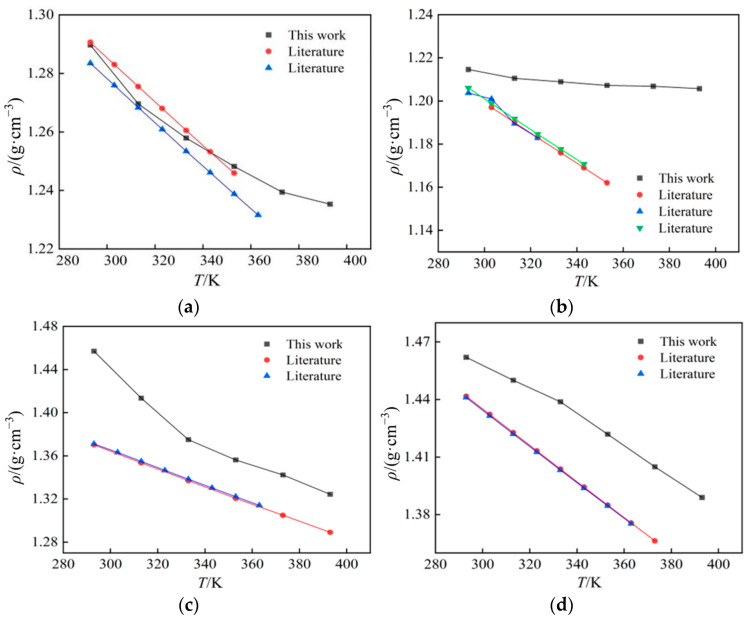
A comparison between computed results and data from the literature of four ionic liquids’ density: (**a**) [Emim][BF_4_]; (**b**) [Bmim][BF_4_]; (**c**) [Bmim][PF_6_]; (**d**) [Bmim][Tf_2_N].

**Figure 3:**
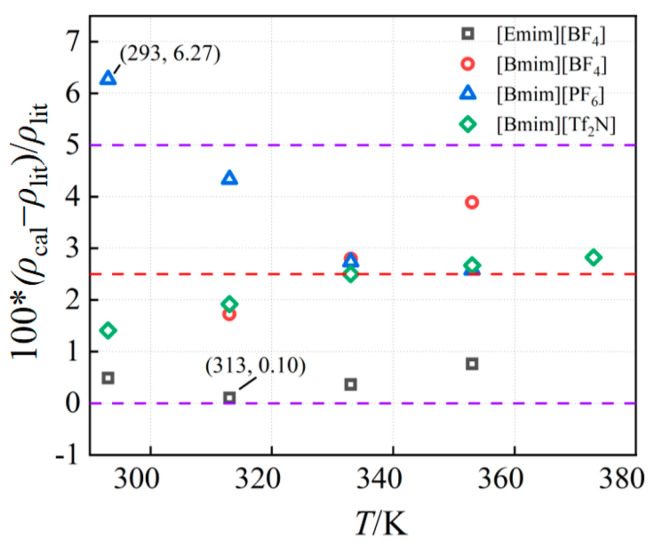
The deviations between computed results and data from the literature.

In this work, the density simulation data of four ionic liquids were compared with the values in the literature [15,16,17,18,19,20,21,22,23], as shown in Figure 2 and Figure 3. Values in the literature were determined by experimental methods, and their values are in general agreement with the trend. The simulated and experimental values are in good agreement, and the relative deviation is mostly within the range of 0~5% at atmospheric pressure, which meets the needs of engineering. The maximum deviation of all comparisons is 6.27% ([Bmim][PF_6_] at 293 K), while the minimum deviation is 0.1% ([Emim][BF_4_] in 313 K).

### 2.2. Viscosity

**Figure 4:**
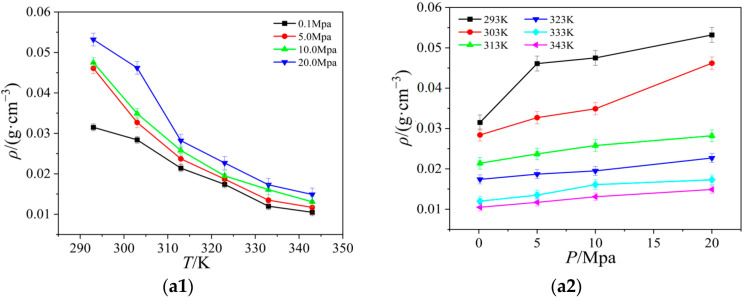

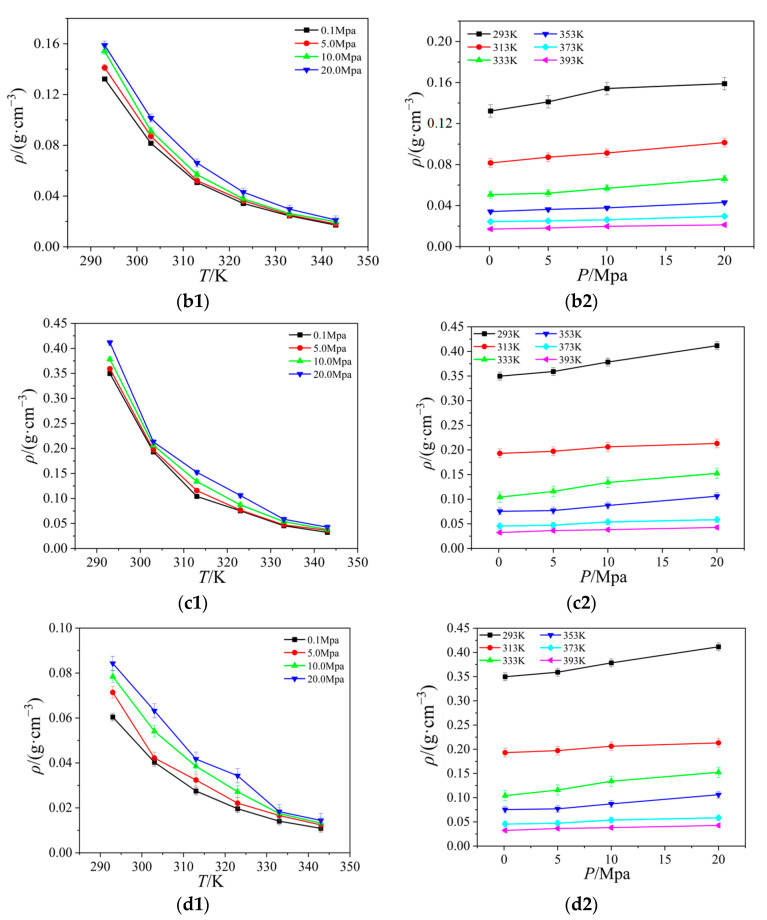
The relationship between the viscosity of four ionic liquids for temperature and pressure: (**a**) [Emim][BF_4_]; (**b**) [Bmim][BF_4_]; (**c**) [Bmim][PF_6_]; (**d**) [Bmim][Tf_2_N].

The accurate prediction of viscosity is important for designing and optimizing various engineering systems and improving productivity. In engineering applications, the correct prediction of viscosity aids in the processing and transportation process. In this section, the viscosity of four ionic liquids at 293 K and at atmospheric pressure was calculated, and compared with the experimental data [22,24,25,26]. Results from the literature show that the magnitude relationship of viscosity between the ionic liquids is [Bmim][PF6] > [Bmim][BF4] > [Bmim][Tf2N] > [Emim][BF4]. This is consistent with the simulation results. The calculated results, the data from the literature, and their relative deviation are listed in Table 1. Obviously, the simulation results agree well with the experimental data, with a maximum relative deviation of −8.96% and a minimum relative deviation of −2.72%. It is adequately demonstrated that the constructed model and the adopted momentum exchange frequency could be used for the simulation of ionic liquid viscosity. Furthermore, simulations of the viscosity of four ionic liquids at different pressures and different temperatures were performed, and the results are shown in Figure 4.

The viscosity of four ionic liquids varies with temperature and pressure. As the tendency displayed in Figure 4 shows, the viscosity fluctuations are primarily caused by temperature changes, and the viscosity reduces with rising temperature and exhibits a nonlinear declining pattern. As the temperature rises, the strength of the change also diminishes, and finally it has a tendency to stabilize. However, the viscosity changes very little and shows no noticeable variations under various pressure situations. It is known that the microscopic movement of the particles is determined by the interactions between the particles. The cations and anions in the ionic liquid gain more kinetic energy as the temperature rises, which speeds up their travel and reduces their interaction. In addition, the adhesive properties of ionic liquid molecules as well as those between cations and anions gradually weakened. Thus, the viscosity steadily drops as temperature rises at a particular shear rate.

### 2.3. Thermal Conductivity

**Figure 5:**
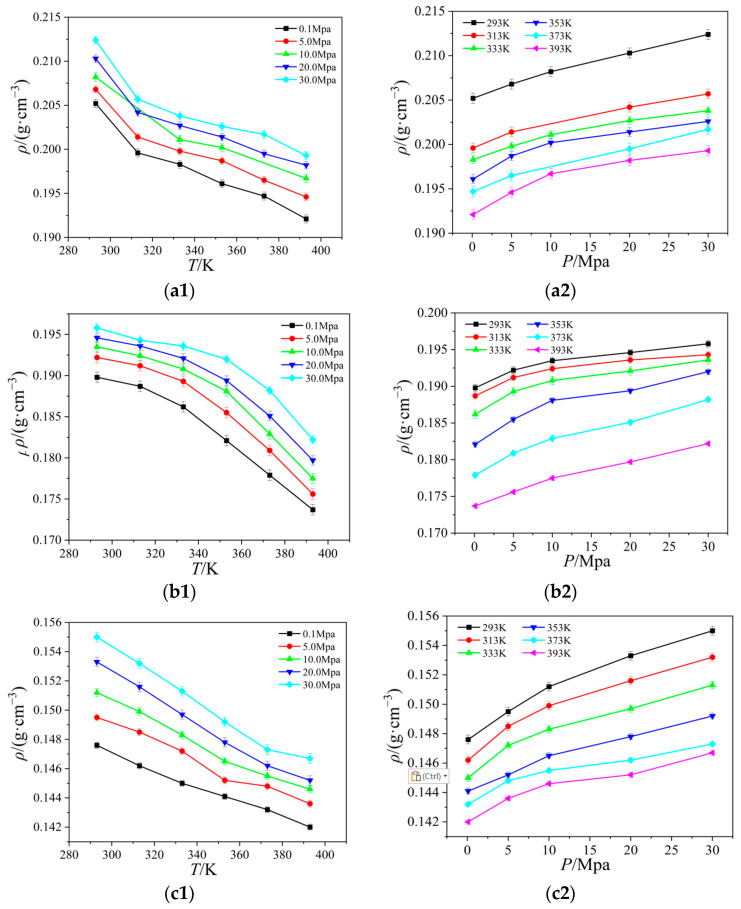

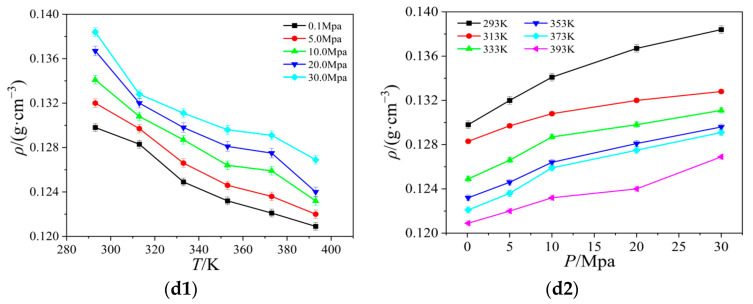
Curves of the thermal conductivity of four ionic liquids with temperature and pressure: (**a**) [Emim][BF_4_]; (**b**) [Bmim][BF_4_]; (**c**) [Bmim][PF_6_]; (**d**) [Bmim][Tf_2_N].

**Figure 6:**
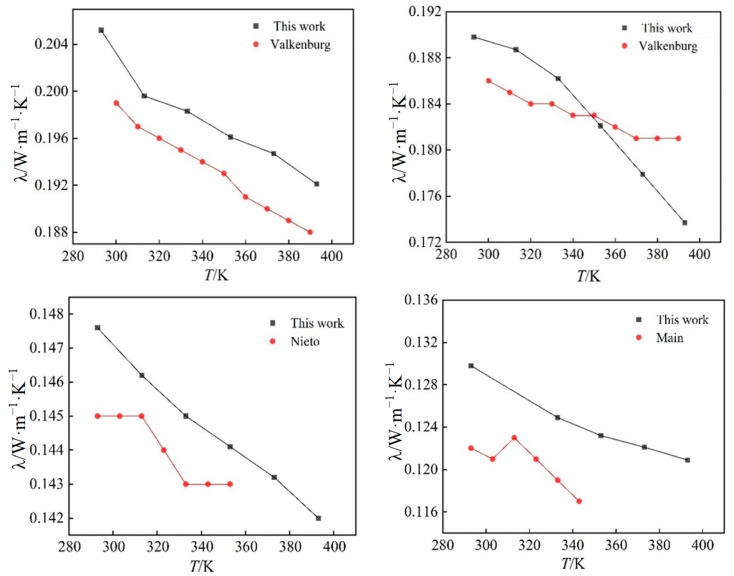
A comparison of simulated and experimental values of thermal conductivity of four ionic liquids at atmospheric pressure.

Thermal conductivity is an important thermophysical parameter, reflecting the thermal performance of the material, and is used to identify the material insulation performance as one of the main signs of good engineering design, and has an important role. In this section, the thermal conductivity values of these four ionic liquids at different pressures and temperatures are calculated with the NEMD method, and the results are shown in Figure 5. At the same time, the simulated values of the thermal conductivity of four ionic liquids at atmospheric pressure are compared with the experimental values in the literature [19,20,21], as shown in Figure 6.

As can be seen from Figure 6, the thermal conductivity curves measured by experimental methods in the literature fluctuate considerably, but in general, the simulated values of the thermal conductivity of the four ionic liquids at atmospheric pressure are in good agreement with the experimental values, and the molecular dynamics of the temperature dependence of the thermal conductivity of the ionic liquids at atmospheric pressure is well simulated. The thermal conductivity of the ionic liquids [Emim][BF_4_], [Bmim][BF_4_], [Bmim][PF_6_], and [BMIM][TF_2_N] decreased with the increase in temperature when the control pressure was constant, and the thermal conductivity of the ionic liquids [Emim][BF_4_], [Bmim][BF_4_], [Bmim][PF_6_], and [Bmim][TF_2_N] all increased with the increase in pressure. The thermal conductivity of ionic liquids is more dependent on temperature, and it changes more obviously with temperature. At the same time, the thermal conductivity of ionic liquids decreases with the increase in the mass of cationic molecules under the same condition of anion [BF_4_]^-^. This phenomenon is due to the extension of the cationic carbon chain, and the increase in the bond length and bond angle; the extension of the bond length, and the bending of the bond angle, restrict the movement of the molecule, resulting in the decrease in the thermal conductivity. When the cation [Bmim]^+^ is the same, with the increase in the number of atoms on anions, the radius of action of anions increases, the distance between clusters between molecules increases, and the number of molecules in unit volume decreases; vibration and heat transfer between ions are weakened, resulting in a decrease in energy transfer between molecules. At the same time, with the increase in temperature, the thermal motion of the molecule becomes stronger and stronger, and the energy loss caused by it becomes larger and larger. Under the combined effect of the two, the heat conduction performance decreases.

## 3. Materials and Methods

### 3.1. The Model of Calculating

**Figure 7:**
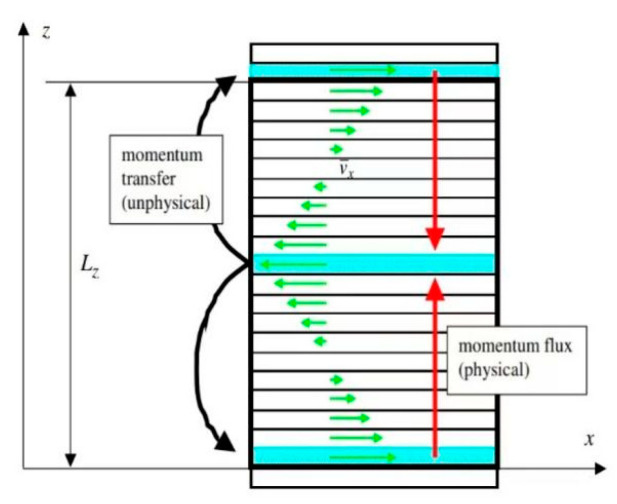
Schematic of the RNEMD method.

**Figure 8:**
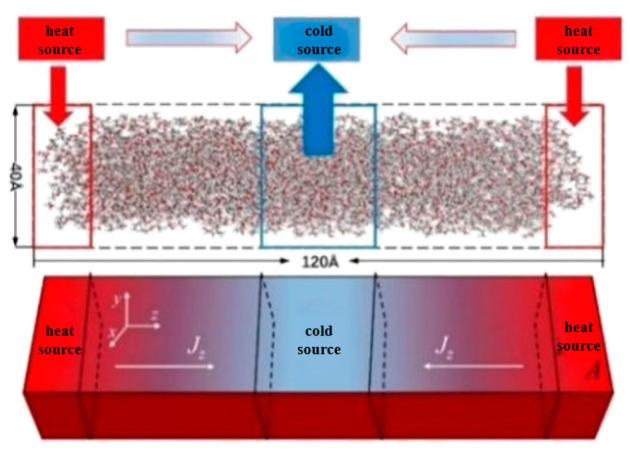
Schematic of the NEMD method.

In this section, the molecular computational models of 1-ethyl-3-methylimidazolium tetrafluoroborate [Emim][BF_4_], 1-butyl-3-methylimidazolium tetrafluoroborate [Bmim][BF_4_], 1-butyl-3-methylimidazolium hexafluorophosphate [Bmim][PF_6_], and 1-butyl-3-methylimidazolium bistrifluoromesulfonimide [Bmim][TF_2_N] were constructed. The model of calculating the density and viscosity is shown in Figure 7. The model of calculating the thermal conductivity is shown in Figure 8. The numbers of [Emim][BF4], [Bmim][BF4], [Bmim][PF6], and [Bmim][Tf2N] molecules within the model are 756, 614, 562, and 397, respectively. All simulations in this paper are performed using the open source software LAMMPS (LAMMPS Molecular Dynamics Simulator) [27] software. 

The length, width, and height of both models are set to 40 Å, 40 Å, and 120 Å. Periodic boundary conditions are adopted in all dimensions to eliminate the boundary effect and keep the particle number constant. The z direction is selected as the transport direction, the whole system is divided into 20 layers along this direction, and the thickness of each layer is 6.0 Å. The van der Waals and Coulomb force cut-off distance is set to 12 Å, which is less than the minimum 1/2 (20 Å) of the box-edge length. If the distance between the two atoms is greater than the truncated radius, then the van der Waals force is approximately zero. For the calculation of long-distance interaction, the PPPM method is adopted, and the precision is set to 1 × 10^−4^. The temperature and pressure of the system are controlled by a Nose–Hoover thermostat and pressure regulator, respectively.

The velocity gradient in the simulation system for density and viscosity is generated by momentum exchange between the particles in layer 1 and 20 and the particles in layer 11. In this paper, the momentum exchange frequency is chosen as every one-thousand steps, according to the test results in the supporting documents, to achieve a good simulation effect. When a liquid flow occurs, the flow rates of its various components are not coordinated, and the pace of the liquid’s movement is conveyed layer by layer. The difference in flow rate is created because the liquid is sticky. The stickiness causes the fluid to divide into different flow layers in the direction of motion. Liquid molecules interact, allowing it to move between the flow layers at varying speeds. This force in a flowing fluid that attempts to lessen the difference in flow velocity is referred to as a viscous force, also known as an internal friction force, and this property of the fluid is referred to as viscosity. The relationship between a fluid’s viscosity and its shear stress and velocity gradient is shown in Formula (1) [28]:

(1) $$\tau =\eta \frac{d\nu }{dz}$$

where *τ* is the shear stress; *η* is the viscosity coefficient; and *dv*/*dz* is the velocity gradient in the *z* direction.

The density of ionic liquid is calculated by the following formula [28]:

(2) $$\rho =\frac{NM}{VN_{A}}$$

where *n* is the number of molecules in the system; *M* is the relative molecular mass; *N_A_* is the Avogadro constant; and *V* is the volume after equilibration.

The momentum exchange and viscosity calculation of the entire system may be described by the following equation once the system’s overall balance has been achieved [28]:

(3) $$p_{\mathrm{transfer}}=\sum (p_{z,1}-p_{z,11})$$

(4) $$\eta =-\frac{p_{\mathrm{transfer}}}{2\Delta tL_{x}L_{y}\langle \partial v_{x}/\partial z\rangle }$$

where *L_x_L_y_* is the area of the section in the *x* direction and *y* direction; *η* represents the viscosity; ∆*t* is the system simulation time; $\partial v_{x}/\partial z$ is the gradient of the velocity in the *x* direction along the *z* direction; and *p*_transfer_ represents the degree of momentum exchange of the system in the *z* direction.

Similar to the density and viscosity calculation system, in the thermal conductivity simulation system, the first layer is set as the heat source, the 11th layer as the cold source, the middle section is the heat transfer region, and the two regions mirror each other. The heat flux of 0.012 kcal/mol was chosen to calculate the thermal conductivity. In the process of simulation, energy is extracted from the cold source while the heat is injected into the heat source to form a temperature gradient. At the beginning of the simulation, the energy of the whole system is minimized to keep the system relaxation balance and avoid the collapse of the simulation system due to atomic overlap and collision. When the system is stable, the temperature and pressure of the NPT ensemble control system remain unchanged for 1 ns. Then, the NPT ensemble is removed, the NVE ensemble is applied to the system, a certain amount of heat flow is added in layer 1, and the same amount of heat flow is extracted from layer 11 to keep the energy of the whole system constant; thus, a temperature field is constructed in the simulation system, and a temperature gradient is formed in the system with a duration of 2 ns. In this process, the thermal and temperature gradients of the system are obtained by analyzing the atomic trajectories, and the thermal conductivity is obtained using Fourier’s law [29]:

(5) $$\lambda =\frac{\sum J}{2\Delta tA_{xy}\langle \partial T/\partial z\rangle }$$

where *j* is the heat flux applied to the system, ∆*t* is the simulated length of time, *A_xy_* is the area of the cross section in the x and y directions, and *<∂T*/*∂z*> is the temperature gradient in the *z* direction.

### 3.2. Simulated Force Field

The OPLS-AA all-atom force field is applicable to the molecular dynamics simulations of the four ionic liquids in this paper [30,31]. The calculated parameters are shown in Table 2. Two sorts of terms—bonded terms and non-bonded terms—are included in the OPLS-AA force field. Bonded terms include bond expansion, angular expansion, dihedral angle, and non-torsion dihedral angle, while non-bonded terms include the short-range Lennard–Jones term and long-term Coulomb term [32,33,34], and the expression of the interacting potential energy function is as follows:

(6) $$E=E_{\mathrm{bonds}}+E_{\mathrm{angles}}+E_{\mathrm{dihedrals}}+E_{\mathrm{impropers}}+E_{\mathrm{nonbond}}+E_{\mathrm{nonbond}}$$

(7) $$E_{\mathrm{bands}}=\sum_{\mathrm{bands}}K_{l}{\left(l-l_{0}\right)}^{2}$$

(8) $$E_{\mathrm{angles}}=\sum_{\mathrm{angles}}K_{\theta }{\left(\theta -\theta_{0}\right)}^{2}$$

(9) $$E_{\mathrm{dihedrals}}=\sum_{\mathrm{dihedrals}}\left\{\frac{1}{2}K_{\varphi 1}\left[1+\cos(\varphi )\right]+\frac{1}{2}K_{\varphi 2}\left[1+\cos(2\varphi )\right]+\frac{1}{2}K_{\varphi 3}\left[1+\cos(3\varphi )\right]\right\}$$

(10) $$E_{\mathrm{impropers}}=\sum_{\mathrm{impropers}}K_{\chi }{\left(\chi -\chi_{0}\right)}^{2}$$

(11) $$E_{\mathrm{nonbond}}=\sum_{i<j}\left\{\frac{Cq_{i}q_{j}}{r_{ij}}+4\varepsilon_{ij}{\left(\frac{\sigma_{ij}}{r_{ij}}\right)}^{12}-{\left(\frac{\sigma_{ij}}{r_{ij}}\right)}^{6}\right\}$$

where *K*_l_, *K*_θ_, *K*_φ_, *K*_χ_, *l*, *θ*, *χ*, and *φ* represent the parameters of bond, angle, dihedral angle, and out-of-plane angle, respectively. In addition, *C* represents the parameter of energy conversion; *q_i_* and *q_j_* represent the amount of charge a particle carries; and *σ_ij_* and *ε_ij_* represent the scale and energy parameters of each atom. The interaction between atoms is characterized by the modified L-J potential function, and the corresponding scale and energy parameters are calculated by the L-J mixing rule, as shown in Equation (12):

(12) $$\begin{array}{c} \sigma_{ij}=\frac{1}{2}(\sigma_{ii}+\sigma_{jj})\\ \varepsilon_{ij}=\sqrt{\varepsilon_{i}\varepsilon_{j}} \end{array}$$

The non-bonding interactions of all atoms are as follows:

## 4. Conclusions

Molecular dynamics simulation was used to simulate the density of four ionic liquids ([Emim][BF_4_], [Bmim][BF_4_], [Bmim][PF_6_], [Bmim][Tf_2_N]) with the pressure ranging from 0.1 MPa to 30.0 MPa and with the temperature ranging from 293 K to 393 K. The computed density is compared to data from the literature; most variances are about 2.5%, and the highest relative deviation is less than 6.27%, indicating a satisfactory level of agreement. Then, the viscosity of four ionic liquids in the temperature range from 293 K to 343 K and under four pressures, 0.1 MPa, 5.0 MPa, 10.0 MPa, and 20.0 Mpa, were simulated using the RNEMD method. According to the findings, ionic liquids’ viscosity steadily decreases as temperature rises, and temperature has a significantly bigger impact on viscosity than pressure. The comparison has also been conducted between the computed results and experimental data at atmospheric pressure and at the temperature 293 K; the maximum relative deviation is −8.96%, and the minimum relative deviation is −2.72%. It was found that the thermal conductivity of ionic liquids decreases with the increase in temperature, and increases with the increase in pressure, and the thermal conductivity of ionic liquids is more sensitive to the change in temperature. Compared with the experimental data of ionic liquids at atmospheric pressure, the simulated thermal conductivity shows good linearity. In this work, the density, viscosity, and thermal conductivity data of four ionic liquids at high pressure was reported for the first time, and it has been proved that molecular dynamics simulation is a feasible way for obtaining the properties of ionic liquids, which is crucial in encouraging their widespread utilization.

## Figures and Tables

**Table 1:** The viscosity of four ionic liquids at 293 K and atmospheric pressure.

Ionic Liquids	[Emim][BF_4_]	[Bmim][BF_4_]	[Bmim][PF_6_]	[Bmim][Tf_2_N]
*η*_cal/_*p*_a_·s	0.0315	0.1323	0.3498	0.0604
*η*_lit*/*_*p*_a_·s	0.0346 [24]	0.1360 [25]	0.3740 [26]	0.0651 [24]
Dev.%	−8.96%	−2.72%	−6.47%	−7.39%

**Table 2:** The non-bonding interactions.

Atomic Type	*q* (e^−^)	*ε* (Kcal/mol)	*σ* (Å)
BF_4_			
B	0.8276	0.095	3.5814
F	−0.4569	0.060	3.1181
PF_6_			
P	1.3400	0.2000	3.7400
F	−0.3900	0.0061	3.1181
NTF_2_			
F	−0.1600	0.0530	2.9500
C	0.3500	0.0660	3.5000
S	1.0200	0.2500	3.5500
O	−0.5300	0.2100	2.9600
N	−0.6600	0.1700	3.2500
EMIM			
CR	−0.09	0.07	3.55
CW	−0.24	0.07	3.55
CW	−0.24	0.07	3.55
HR	0.21	0.03	2.42
HW	0.27	0.03	2.42
HW	0.27	0.03	2.42
CM	−0.35	0.066	3.55
HM	0.18	0.03	2.55
HM	0.18	0.03	2.55
HM	0.18	0.03	2.55
NA	0.22	0.17	3.25
CA	−0.17	0.066	3.5
HA	0.18	0.03	2.5
CT	−0.24	0.066	3.5
HT	0.08	0.03	2.5
HT	0.08	0.03	2.5
HT	0.08	0.03	2.5
NA	0.22	0.17	3.25
HA	0.18	0.03	2.5
BMIM			
NA	0.22	0.17	3.25
NA	0.22	0.17	3.25
CR	−0.09	0.07	3.55
HR	0.21	0.03	2.42
CW	−0.24	0.07	3.55
HW	0.27	0.03	2.42
CW	−0.24	0.07	3.55
HW	0.27	0.03	2.42
CM	−0.35	0.066	3.5
HM	0.18	0.03	2.42
HM	0.18	0.03	2.42
HM	0.18	0.03	2.42
CA	−0.17	0.066	3.5
HA	0.18	0.03	2.42
HA	0.18	0.03	2.42
CS	−0.12	0.066	3.5
HS	0.06	0.03	2.42
HS	0.06	0.03	2.42
CS	−0.12	0.066	3.5
HS	0.06	0.03	2.42
HS	0.06	0.03	2.42
CT	−0.24	0.066	3.5
HT	0.08	0.03	2.42
HT	0.08	0.03	2.42

## Data Availability

Data are contained within the article.
